# The effect of moderate-intensity exercises on physical fitness, adiposity, and cardiovascular risk factors in Saudi males university students

**DOI:** 10.25122/jml-2023-0018

**Published:** 2023-05

**Authors:** Said El-Ashker, Mohammed Al-Hariri

**Affiliations:** 1. Self-Development Department, Deanship of Preparatory Year, Imam Abdulrahman Bin Faisal University, Dammam, Saudi Arabia; 2. Department of Physiology, College of Medicine, Imam Abdulrahman Bin Faisal University, Dammam, Saudi Arabia

**Keywords:** BMI, adiposity, physical activity, cardiovascular risk, VO_2_ max

## Abstract

Physical inactivity has been linked to several non-communicable diseases. This cross-sectional study investigated the impact of moderate-intensity exercise on physical fitness, adiposity, and cardiovascular risk factors in 284 Saudi male university students in the Eastern Province of Saudi Arabia between 2017 and 2019. The physical activity (PA) intervention comprised three (120 minutes each) sessions of moderate intensity on non-consecutive days each week, delivering a total of 360 minutes of PA per week for ten weeks. We measured physical fitness using VO_2_ max calculations and assessed adiposity and cardiovascular risk factors using a range of parameters, such as systolic/diastolic blood pressure, heart rate, mean arterial pressure, body mass index, waist circumference, waist-to-height ratio, waist-to-hip ratio, body adiposity index, and body fat percentage. Our study found a significant increase in VO_2_ max after ten weeks of moderate-intensity exercise (<0.001). Additionally, body adiposity indices were significantly reduced before and after the intervention (p<0.001), as were cardiovascular risk factors. Our findings suggest that regular moderate-intensity exercise effectively improves physical fitness, decreases adiposity, and lowers cardiovascular risk factors in Saudi male adults. We recommend that policymakers and public health practitioners increase physical activity among university students by launching a campaign on social media and through

## INTRODUCTION

Physical inactivity has been linked to a number of non-communicable diseases, including obesity, diabetes, chronic respiratory disease, and cardiovascular disease [1]. According to a report in 2016, physical inactivity caused 7.1% of breast cancer, 7.0% of colon cancer, 4.9% of type 2 diabetes, 4.5% of stroke, 4.0% of coronary heart diseases, and 6.4% of all-cause mortality [2]. Alarmingly, the number of physically inactive individuals has continuously increased from 14.4 million to 48.6 million between 1995 and 2020, representing a 240% increase [3]. Despite significant efforts to promote physical activity as a health behavior, 27.5% of individuals still do not meet the recommended levels of physical activity according to current public health guidelines [4].

According to international health recommendations, youth should complete at least 60 minutes of moderate to vigorous physical activity daily [5]. However, published evidence indicates that contemporary adults are not as active as they should be [6,7]. In fact, recent international data indicate that most adult male university students are physically inactive [8–10].

During the past several decades, Saudi Arabia has witnessed significant economic growth and technological transformation, resulting in dramatic lifestyle changes [11]. Consequently, sedentary behaviors, physical inactivity, and consumption of sugar-sweetened beverages, as well as a high caloric-dense diet, became prevalent in Saudis, which contributed to an increase in the prevalence of non-communicable diseases such as dyslipidemia, obesity, lipotoxicity, diabetes mellitus, hypertension, cardio, and coronary artery diseases [12–15].

Regular exercise and physical activity have been directly linked to lower mortality and morbidity rates [16]. However, while the effects of exercise training on the cardiovascular system have been extensively studied, most of these studies have focused on men or young volunteers. In addition, health and medical organizations stress the importance of providing accurate information on optimal exercise intensity and volume for physical activity and exercise professionals to achieve the desired effectiveness[17].

Several reports have indicated a high prevalence of physical inactivity among adults in Saudi Arabia, making it challenging for them to achieve the health benefits of physical activity[12,18]. However, in the past decade, there have been published reports regarding physical activity among young adults, highlighting the need for an update on exercise interventions in this rapidly growing Middle Eastern country. Currently, limited and scant data is available in the published literature on the effectiveness of moderate exercise or PA among Saudi university students. Therefore, this study aimed to investigate the effects of a ten-week moderate-intensity exercise intervention, consisting of a convenient bout, on adiposity, physical fitness, and cardiovascular risk factors in male Saudi students.

## MATERIAL AND METHODS

### Study design and setting

A structured cross-sectional study was carried out from 2017 to 2019 in the Eastern Province of Saudi Arabia among male Saudi adult students. The study recruited participants (N=1350) from the university community through flyers; any student who could exercise was eligible to participate. Participants with a history of medical issues (e.g., endocrine, respiratory, cardiac, renal, malignancy) or joint pain and smoking were excluded from the study. Each student completed a medical history questionnaire to ensure consistency with their physical activity activities.

### Data collection tools

The obesity and cardiovascular parameters were measured using standardized equipment by qualified research assistants according to international standards and references. The weight of the students was documented to the nearest 0.1 kg and height to the nearest 0.1 cm (Seca 704; Seca, Hamburg, Germany) [19]. Other measurements, such as waist circumference (WC) and hip circumference (HC), were obtained using a measuring tape to the nearest centimeter, as these are commonly used non-invasive biomarkers for predicting cardio-metabolic risk factors [20].

Two consecutive readings of systolic and diastolic blood pressure (BP) were taken from each participant after 15 min of resting using an automated blood pressure monitor (Omron M6 Comfort IT) [21]. Digital handgrip dynamometry (Product of SAEHAN Corporation Company, South Korea) was used to test the right-left-hand grip. The instrument was considered reliable based on the evidence provided by Faria in 2013 [22]. All measurements were taken between 11:00 AM and 12:30 PM to control for confounders and diurnal hormonal variations on the recorded results [23]. To ensure the utmost reliability of the collected data, participants were asked to press the dynamometer two times with a one-minute rest between each recording, and the mean of three records was considered for data analysis.

### Indices calculations

Body mass index (BMI) was calculated by dividing the participant's weight in kilograms by the square of their height in meters [BMI=Weight (kg)/Height (m)2] [24]. Based on the BMI measurements, students were categorized as normal weight (5–85th percentile), overweight (85–95th percentile), and obese (>95th percentile) [25].

The body adiposity index (BAI) was calculated as BAI=((hip circumference)/((height)1.5)–18)) [26]. Basal metabolic rate (BMR) = [(10 x weight in kg) + (6.25 x height in cm) - (5 x age in years) + 5] Kcal/day [27]. We calculated the waist-to-hip ratio by dividing waist circumference/hip circumference. The waist-to-height ratio was calculated as waist circumference/height [28]. The VO_2_ max was calculated using the Uth N. (2004) formula indirectly from the measured HR(max)-to-HR(rest) ratio [29]. Mean arterial pressure was calculated using diastolic pressure + 1/3 pulse pressure [30].

### Physical activity intervention

The intervention protocol consisted of three sessions per week, each lasting 120 minutes on non-consecutive days, for a total of 360 minutes per week, for ten weeks. The intensity of the physical activity (PA) was managed by maintaining the target heart rate in the moderate-intensity zone (64% to 76% of the maximum heart rate) [31]. The physical education lesson activities were graded to achieve a load intensity that permitted the participants to sustain a perceived exertion (RPE) of 12 to 14 on the Borg 6-20 point scale [32]. RPE and target heart rate were recorded every 10 minutes during the activities.

The university provided physical education lessons (120 minutes/week) to all students. The study participants were attending these physical education lessons compulsory, and the PA sessions were conducted under the supervision of the same physical education instructors. A prerequisite for participation in the study was to attend at least 90% of the sessions. The applied intervention protocol was based on the United States Centres for Disease Control and Prevention guidelines [33] to achieve 150 minutes of moderate-intensity PA plus another two days of muscle-strengthening activity.

The intervention protocol integrated activities preferred by the study participants based on their responses to the favorite sport type in the PA readiness questionnaire (240 minutes per week). Accordingly, the intervention was composed of outdoor activities such as football (n=60, 21.13%), volleyball (n=46, 16.20%), handball (n=37, 13.03%), basketball (n=32, 11.27%), and running (n=25, 8.80%), as well as indoor activities (table tennis n=34, 11.97%), gym (n=28, 9.86%), and squash (n=22, 7.75%) as summarized in Table 1.

**Table 1.** Exercise protocol for supervised physical activity intervention workouts

**Frequency**	2 controlled physical education lessons per week for 8 weeks
**Intensity**	Moderate intensity was prescribed for the exercise sessions, and the students were instructed to perform the exercises at a level where they could breathe moderately, experience flushed skin and sweat.
**Time**	120 minutes consist of: 10 minutes workout introductory session (e.g., attendance, goals); 20 minutes warm-up; 40 minutes of selected sport activity; 20 minutes of skill-related physical fitness exercises, 2 components for each workout (e.g., balance, speed, agility, coordination); 20 minutes of health-related physical fitness exercises, 2 components for each workout (e.g., cardiovascular endurance, strength, flexibility, muscular endurance; 10 minutes cool-down.
**Type**	Activities included a range of outdoor activities (e.g., football, volleyball, handball, basketball, and running), indoor activities (e.g., table tennis, gym, and squash), and physical fitness exercise.

### Statistical analysis

Statistical analysis was performed using SPSS software (Version 24, Chicago, IL, USA). Descriptive statistics were calculated for all baseline and post-intervention variables, including the mean and standard deviation (mean ± SD). Pairwise t-tests were used to determine significant differences between baseline and post-intervention variables. All p-values were two-tailed, and significance was considered at a level of <0.05.

## RESULTS

### Baseline characteristics

**Figure 1.**
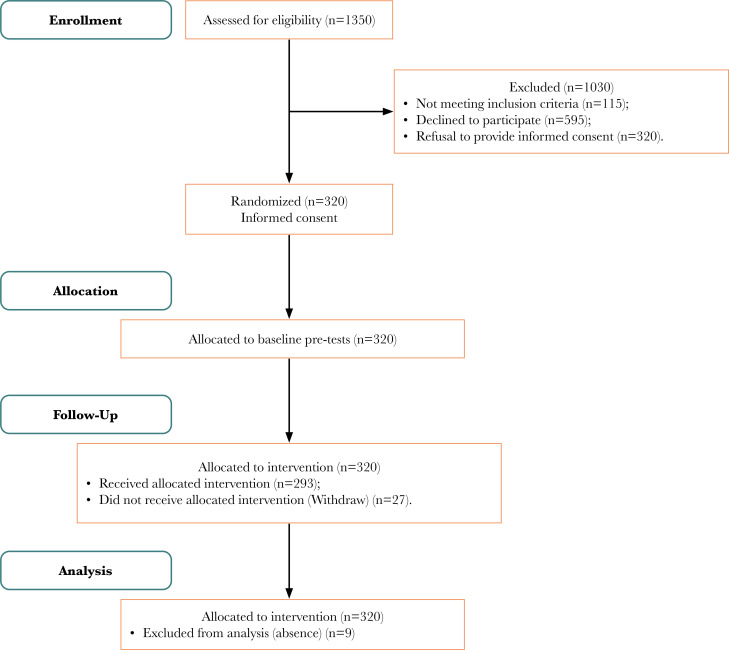
Flowchart illustrating the selection process of students for the physical activity intervention

A total of 320 students initially responded and agreed to participate in the study. However, 36 students were excluded from the final analysis because they could not attend all measurement sessions or post-tests in person. Therefore, a convenience sample of 284 male Saudi students who completed all the intended measures before and after the ten-week PA intervention was included in the analysis (see Figure 1). The mean age of participants was 18.5±0.9 years, the mean height was 176±8.3 cm, and the mean weight was 84.9±30.6 kg. The mean heart rate, systolic and diastolic blood pressure were 80.0±12.05 bpm, 127.5±14.5 mmHg, and 70.6±9.7 mmHg, respectively (Table 2).

**Table 2.** Baseline characteristics of participants

Characteristics (n=284)	Mean±SD
**Age**	18.5±0.9
**Height (cm)**	176±8.3
**Body weight (kg)**	84.9±30.6
**Heart rate (BPM)**	80.0±12.05
**Systolic blood pressure (mmHg)**	127.5±14.5
**Diastolic blood pressure (mmHg)**	70.6±9.7

BPM – beat per minute.

### Cardiovascular indices

As illustrated in Table 3, performing weekly PA bouts or each exercise session showed a significant reduction in the cardiovascular indices as recorded at the end of the exercise protocol (ten weeks) compared to baseline. Resting heart rate significantly decreased (p<0.001) from 80.0±12.05 to 73.7±10.11 beats per minute. Systolic and diastolic blood pressure also significantly decreased (p=0.005, 0.001 respectively) from 127.5±14.53 and 70.6±9.76 mmHg to 126.0±6.98 and 69.1±3.84 mmHg, respectively. Furthermore, the mean arterial pressure, which represents the driving force of blood flow, significantly decreased (p≤0.001) from 89.6±9.53 to 88.1±4.07 mmHg after the exercise intervention (Table 3).

**Table 3.** Cardiovascular indices before and after the intervention

Parameter	Mean±SD		P-value
Preintervention reading	Postintervention reading	
**Heart rate (BPM)**	80.0±12.05	73.7±10.11	<0.001
**Systolic blood pressure (mmHg)**	127.5±14.53	126.0±6.98	0.005
**Diastolic blood pressure (mmHg)**	70.6±9.76	69.1±3.84	0.001
**Mean arterial pressure**	89.6±9.53	88.1±4.07	<0.001

BPM – beat per minute.

### Body adiposity indices and basal metabolic rate

There were significant improvements (p<0.001) in all of the study body adiposity and composition indices before and after the completion of the exercise program. After ten weeks of convenient PA bouts, waist circumference significantly (p<0.001) decreased from 93.8±21.67 cm to 84.8±17.59 cm, and both the calculated waist-to-hip ratio and waist-to-height ratio significantly (p<0.001) decreased from 0.9±0.084 and 0.5±0.120 to 0.85±0.09 and 0.46±0.09, respectively. Similarly, significant improvements (p<0.001) were shown in body adiposity index, body fat percentage, body mass index, and basal metabolic rate 28.1±7.62, 31.6±11.19%, 27.3±9.32 kg/m^2^, 1863.1±423.47 Kcal/day compared to baseline 24.0±5.84, 30.6±10.41%, 26.4± 8.67 kg/m^2^, 1835.3±303.30 Kcal/day, respectively (Table 4).

**Table 4.** Body adiposity indices and basal metabolic rates before and after the intervention

Parameter	Mean±SD	P-value
Preintervention reading	Postintervention reading	
**Waist circumference (cm)**	93.8±21.67	84.8±17.59	<0.001
**Waist-to-hip ratio**	0.9±0.084	0.85±0.09	<0.001
**Waist-to-height ratio**	0.5±0.12	0.46±0.09	<0.001
**Body adiposity index**	28.1±7.62	24.0±5.84	<0.001
**Body fat percentage (%)**	31.6±11.19	30.6±10.41	<0.001
**Body mass index (kg/m^2^)**	27.3±9.32	26.4±8.67	<0.001
**Basal metabolic rate (Kcal/day)**	1863.1±423.47	1835.3±303.30	<0.001

### Fitness and muscle performance

**Figure 2.**
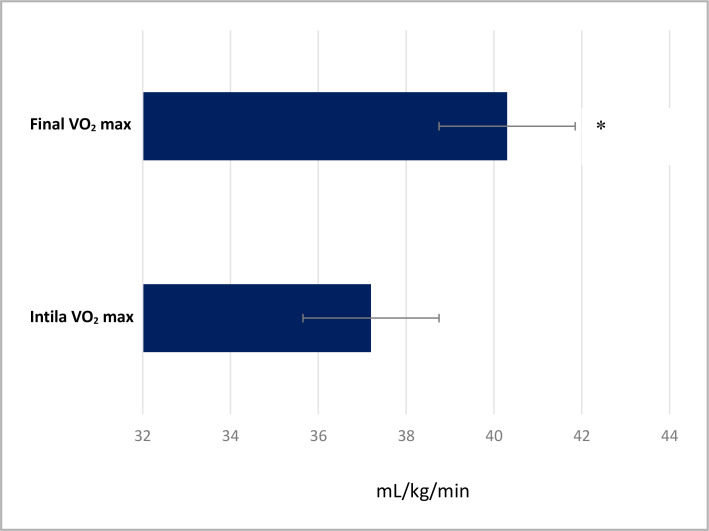
Changes in the calculated VO_2_ max, before and after the intervention

**Figure 3.**
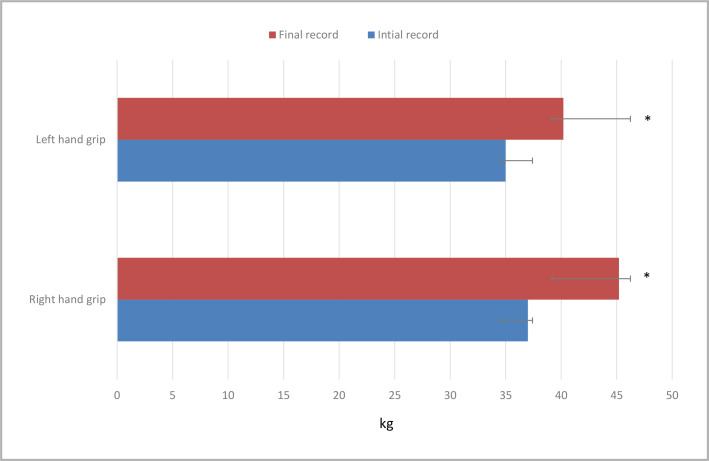
Changes in muscle performance before and after the intervention

Interestingly, our study showed that the calculated VO_2_ max significantly increased (p<0.001) from 37.2±6.3 mL/kg/min to 40.3±6.2 mL/kg/min after ten weeks (Figure 2). In addition, hand grip strength tests of both hands showed a significant (p<0.001) improvement before and after the exercise intervention, with right-hand grip increasing from 37.0±7.31 kg to 45.2±6.67 kg and left-hand grip increasing from 35.0±7.29 kg to 40.2±7.15 kg (Figure 3).

## DISCUSSION

The results of this study demonstrate the positive impact of a ten-week moderate-intensity exercise program on physical fitness, adiposity, and cardiovascular risk factors in male Saudi university students. Physical inactivity is currently considered one of the most significant public health concerns of the 21st century [34]. Unfortunately, physical education and activity are underappreciated and undervalued by many academic institutions and even clinical medicine. Studies conducted among university students in different countries have reported that physical activity levels are often below the recommended guidelines [10,35].

It is widely recognized that prolonged exercise training can elicit numerous physiological adaptations. However, maintaining long-term compliance with exercise programs remains challenging for many individuals [36]. Nevertheless, with sufficient and appropriate exercise stimuli, almost all trainees can benefit from exercise protocols [36]. To address this issue, we implemented a 10-week moderate-intensity exercise program consisting of convenient and enjoyable modalities to investigate its impact on various physiological parameters among young male participants.

Combination interventions, including both aerobic exercise and resistance training, have been shown to have better outcomes in reducing total body fat, visceral fat, and fat percentage than aerobic exercise alone, according to previously reported data [37,38]. Higher levels of adiposity have been linked to an increased risk of developing various serious illnesses such as hypertension, type 2 diabetes, and cardiovascular and metabolic diseases[39]. Therefore, using convenient exercise programs can be an effective means of reducing this risk.

Low cardiorespiratory fitness is a significant risk factor for mortality, accounting for 16% of all deaths, compared to other traditional causes of death such as smoking, obesity, high cholesterol, diabetes, and hypertension [34]. Moreover, an increase of one metabolic equivalent in cardiorespiratory fitness led to an 18% decrease in deaths due to cardiovascular disease. The current improvement in calculated VO_2_ max falls within the range of what has been previously reported [40]. These improvements have important long-term prevention implications against cardiovascular-metabolic disorders.

The post-training VO_2_ max record obtained in our study and across several studies could be attributable to the completion and compliance rather than the type of exercise. In this regard, it has been reported that a relatively prolonged regimen of moderate or even more intense intervals of PA induces similar improvements in cardiorespiratory fitness [41]. The significant increase in hand grip strength observed in our study after ten weeks is another advantage of utilizing this exercise program. These results confirm the well-known benefits of regular physical activity on muscular strength, independent of an individual's perceived health status [9,42].

Unfortunately, sports courses and physical education are often undervalued and neglected, and administrative, systemic, and financial constraints hinder the development of a strong sports culture. Moreover, student involvement in physical activity is often low, estimated to be only 39%, according to a recent report [43]. Therefore, it is crucial to adopt feasible physical activity models among the younger generation and to encourage partial engagement in academic and physical education to prevent the risk of cardiovascular and metabolic disorders and to improve cardiopulmonary fitness and muscle strength.

One limitation of this study is that it was conducted among male university students using a convenient sampling technique, which limits the generalizability of the results to the broader population. Additionally, the study did not measure biochemical markers such as glucose and lipid profiles to assess the impact of the exercise program on the metabolic status of university students. Furthermore, the study did not investigate the challenges in performing physical activity, and as such, further research is recommended to identify the barriers that prevent students from engaging in regular physical activity.

Based on our findings, we recommend that public health practitioners and policymakers launch an awareness campaign through flyers and social media to encourage adult students to engage in regular physical activity, both on campus and at home. Future studies should aim to overcome the limitations of this research by increasing the sample size, including female participants, and incorporating additional variables such as biochemical markers of metabolic health.

## CONCLUSION

The present study highlights the importance of regular moderate-intensity exercise in improving physical fitness and reducing adiposity and cardiovascular risk factors in Saudi male adults.
